# A randomised, double-blind, placebo-controlled trial of minocycline and/or omega-3 fatty acids added to treatment as usual for at-risk mental states (NAYAB): study protocol

**DOI:** 10.1186/s13063-017-2275-y

**Published:** 2017-11-09

**Authors:** Inti Qurashi, Imran B. Chaudhry, Ameer B. Khoso, Sana Farooque, Steve Lane, Mohammad Omair Husain, Simon Chu, Jane Sarginson, Munir Hamarani, Haider A. Naqvi, Bushra Razzaque, Fareed A. Minhas, Alison R. Yung, J. F. W. Deakin, Nusrat Husain

**Affiliations:** 1 Ashworth Research Centre, Mersey Care NHS Foundation Trust, Maghull, UK; 2 Faculty of Medicine, Biology and Health, University of Manchester, Manchester, UK; 3 Pakistan Institute of Living & Learning, Karachi, Pakistan; 4 Institute of Translational Medicine, University of Liverpool, Liverpool, UK; 5 Manchester Mental Health and Social Care Trust, Manchester, UK; 6 School of Psychology, University of Central Lancashire, Preston, UK; 7 Abbasi Shaheed Hospital, Karachi, Pakistan; 8 Ziauddin University, Karachi, Pakistan; 9 Institute of Psychiatry & WHO Collaborating Centre, Rawalpindi, Pakistan

**Keywords:** Minocycline, Omega-3 fatty acids, PUFA, At-risk mental state (ARMS), Ultra-high risk (UHR) schizophrenia, Global mental health

## Abstract

**Background:**

The at-risk mental state (ARMS) describes individuals at high risk of developing schizophrenia or psychosis. The use of antipsychotics in this population is not supported, because most individuals with ARMS are unlikely to develop psychosis. Anti-inflammatory treatments and polyunsaturated fatty acids (PUFAs) may have some beneficial effects in the treatment of ARMS. There have been no controlled clinical trials in which researchers have investigated the use of minocycline for ARMS and no trials involving PUFAs in combination with other proposed treatments. There is a need to find effective, tolerable and inexpensive interventions for individuals with ARMS that are available in high-, low- and middle-income countries.

**Methods/design:**

A 6-month intervention study of minocycline and/or omega-3 fatty acids added to treatment as usual (TAU) in patients with ARMS will be conducted in Pakistan using a randomised, placebo-controlled, double-blind factorial design. A total of 320 consenting patients with capacity will be recruited from the community, general practitioner clinics and psychiatric units. Allowing for a 25% dropout rate, we will recruit 59 completing participants into each study arm, and in total 236 will complete the study. We will determine whether the addition of minocycline and/or omega-3 fatty acids to TAU attenuates the rate of transition from ARMS to first-episode psychosis and improves symptoms and/or level of functioning in ARMS. We will also investigate whether any candidate risk factors, such as negative symptoms, influence treatment response in the ARMS group. The primary efficacy endpoint is conversion to psychotic disorder at 12 months after study entry. Analysis will be done according to the intention to treat principle using analysis of variance, chi-square tests and adjusted ORs to assess between-group differences. Cox regression analysis will be used to evaluate potential between-group differences in time to onset of psychosis.

**Discussion:**

The outcomes of this trial will provide evidence of the potential benefits of minocycline and PUFAs in the treatment of ARMS. Both minocycline and PUFAs are inexpensive, are readily available in low-/middle-income countries such as Pakistan, and if proven, may be safe and effective for treating individuals with ARMS.

**Trial registration:**

ClinicalTrials.gov, NCT02569307. Registered on 3 October 2015.

**Electronic supplementary material:**

The online version of this article (doi:10.1186/s13063-017-2275-y) contains supplementary material, which is available to authorized users.

## Background

The at-risk mental state (ARMS) describes individuals at increased risk of developing psychosis. Operationalised criteria have been developed to identify young persons with ARMS [1]. Authors of a meta-analysis of 27 studies with a combined sample of 2500 subjects reported a transition to psychosis rate of 18% at 6 months, rising to 22% at 1 year [2]. These studies were undertaken in Europe, the United States and Australia, and transition rates are likely to be higher, approaching 40%, in countries with less-established mental health care systems [3]. Individuals who experience symptoms of ARMS are mostly help-seeking [4], making it a critical period during which an intervention could be delivered to prevent conversion to psychosis, improve symptoms and reduce distress. Most of the disability develops before the onset of frank psychosis [5], and earlier treatment of psychosis is linked to better outcomes [6].

Interventions tested for ARMS include antipsychotics [7] and cognitive behavioural therapy (CBT) [8]. The use of antipsychotics in this population is not supported in, for example, national clinical guidance [9], because a large proportion of individuals with ARMS are unlikely to develop psychosis. Furthermore, antipsychotics can cause side effects such as weight gain and other metabolic disturbances. An alternative approach is to explore the effectiveness of interventions within a clinically staged approach where the earlier in the course of illness treatment is offered, the safer and more tolerable it should be compared with treatments used later in the illness, whilst remaining effective in terms of remission and recovery rates [10].

There is persuasive evidence to test the use of minocycline in individuals with ARMS. Authors of a meta-analysis of minocycline augmentation of antipsychotics pooled the results of four randomised controlled trials including young adults in the early phase of schizophrenia [11] and found that minocycline was superior to placebo in reducing total scores on the Positive and Negative Syndrome Scale (PANSS), the PANSS negative subscale, and the PANSS general subscale. Minocycline was equivalent to placebo for all-cause discontinuation and for discontinuation because of adverse events. In addition, minocycline has a well-established effectiveness and safety profile in adolescents and young adults [12].

Authors of three systematic reviews [13–15] of interventions in the ARMS group found that antipsychotics, CBT and omega-3 fatty acids may have some effect on preventing or delaying the onset of psychosis, but all of these authors noted the need for ongoing research. A replication study of omega-3 fatty acids with a larger sample would be especially important, given that this treatment is relatively safe and has few health risks in a drug-naïve population that could gain other potential benefits (such as improving cardiovascular status). In a subsequent study to determine whether omega-3 fatty acids in combination with a high-quality psychosocial intervention, cognitive behavioural case management (CBCM), was more effective than placebo plus CBCM, researchers did not detect a difference in transition to psychosis at 6 months [16]. However, the lower-than-expected transition rate may have prevented a test of the main hypothesis, and the substantial symptomatic and functional improvement in both groups with the other treatments received (i.e., CBCM and antidepressants) is likely to have produced a ceiling effect beyond which omega-3 fatty acids, even if effective, could not be shown to confer additional benefits. Neither CBT nor CBCM is widely available in low- and middle-income countries, unlike nutritional supplements and antibiotics.

In summary, there is a need to find effective, tolerable, inexpensive and safe interventions for individuals with ARMS, especially in view of the lack of availability of high-quality psychosocial interventions in low-/middle-income countries. Using a factorial design, we will compare placebo with minocycline and omega-3 fatty acids alone, as well as in combination, in the treatment of individuals with ARMS in Pakistan. The novel aspects of this trial include that (1) minocycline has not been tested as a treatment for individuals with ARMS, (2) minocycline and omega-3 fatty acids have not previously been tested in combination and potentially have additive/synergistic effects due to their modes of action, and (3) no drug trials for ARMS have been undertaken in Pakistan or similar low- and middle-income countries.

## Methods/design

### Objectives

The aim of the study is to evaluate the efficacy and tolerability of minocycline and omega-3 fatty acids in individuals with ARMS and specifically to determine whether the addition of minocycline and/or omega-3 fatty acids to treatment as usual (TAU) in an operationalised ARMS population in Pakistan (1) attenuates the rate or incidence of transition from ARMS to first-episode psychosis and (2) improves symptoms and/or level of functioning of individuals with ARMS. In addition, we will explore whether candidate risk factors, such as negative symptoms, socio-demographic characteristics and neurobiological variables, including metabolic parameters (such as erythrocyte membrane fatty acids and, where possible, cytokines), influence the transition to psychosis in the ARMS group.

### Study design

We are conducting a 6-month intervention study of minocycline and/or omega-3 fatty acids added to TAU in patients with ARMS, using a randomised, placebo-controlled, double-blind factorial design. In practice, TAU in this population in Pakistan comprises no specific treatment, whether psychological or pharmacological. Participants will be randomly assigned to either (1) placebo, (2) minocycline, (3) omega-3 fatty acids or (4) minocycline and omega-3 fatty acids in combination. Blinding will be maintained because participants will receive equivalent numbers of tablets. In a factorial design the objective is generally not to compare arms with each other; instead, combinations of experimental conditions are compared. Each experimental condition in a factorial design represents a different treatment protocol. It allows a test of the possibility that the interventions being tested have additive or multiplicative benefits.

### Primary outcome measure

The primary efficacy endpoint will be transition to psychotic disorder at 12 months after study entry. Transition is operationally defined using the positive symptom subscales in the Comprehensive Assessment of At Risk Mental States (CAARMS) [17] as one of the following:

- Unusual thought content held with delusional intensity (global score 6) occurring several times or more per week (frequency and duration score > 3)
- Non-bizarre ideas held with delusional conviction (global score 6) occurring several times or more per week (frequency and duration score > 3)
- Perceptual abnormalities in any modality (global score ≥ 5) occurring several times or more per week (frequency and duration score > 3)
- Disorganised speech (global score ≥ 5) occurring several times or more per week (frequency and duration score > 4)

### Secondary outcome measures

Secondary outcome measures are as follows:

- Change in severity of ARMS symptoms between entry (baseline) assessment and at 3, 6 and 12 months measured as difference in scores on the CAARMS
- Change in social and occupational functioning between baseline and 3, 6 and 12 months measured using the Social and Occupational Functioning Assessment Scale (SOFAS) [18]
- Change in cognitive scores between baseline and 3, 6 and 12 months measured using the CogState battery [19]
- Adverse effects of medications: proportion developing adverse effects as a function of severity
- Treatment with antipsychotic medication (from participant self-report and subsequent confirmation by clinician or by prescription evidence), because this is likely to correlate with conversion to psychosis
- Change in severity of depressive symptoms between entry at baseline and at 3, 6 and 12 months measured as the difference in scores on the Montgomery-Åsberg Depression Rating Scale (MADRS) [20]

### Sample size

Sample size estimates are based on the expected transition rate to psychosis of 30%, and, on the basis of previous research, we expect to achieve a clinically meaningful reduction to 15% [21, 22] following treatment with minocycline and omega-3 fatty acids. With 80% power and at a 5% significance level, we will require a sample size of 59 per treatment arm to detect the expected difference. We are allowing for a drop-out rate of 25%, and in order to maintain 59 completing participants per arm, we will recruit 80 participants to each of the 4 trial arms. Therefore, we intend to recruit 320 participants in total.

### Study participants

#### Inclusion criteria

Participants should fulfil all of the following criteria:

- Male or female individuals aged 16–35 years
- Meet the criteria for ARMS using CAARMS operationalised intake criteria based on three groups (vulnerability, attenuated psychosis, or brief limited intermittent psychotic symptoms group)
- Assessed as competent to provide informed consent

#### Exclusion criteria

Individuals meeting any of the following criteria will be excluded:

- A history of psychotic illness (treated or untreated)
- IQ < 70 and/or a history of learning disability
- Any pre-existing inflammatory conditions (e.g., rheumatoid arthritis)
- Organic brain disease (e.g., epilepsy)
- Previous treatment with an antipsychotic or mood-stabilising agent
- Prior history of intolerance or serious side effects (hepatotoxicity, photosensitivity, blood dyscrasia) of any of the tetracyclines or omega-3 fatty acids
- Concomitant penicillin therapy or concomitant anticoagulant therapy
- Active substance abuse (except nicotine or caffeine) or dependence within the last 3 months, according to *Diagnostic and Statistical Manual of Mental Disorders, Fifth Edition*, criteria
- Treatment with warfarin or lamotrigine
- Current or previous treatment with tetracycline antibiotics or omega-3 fatty acids in the 3 months before study entry
- Current treatment with any anti-inflammatory medication
- Treatment with electroconvulsive therapy within the 12 weeks preceding the study
- Active expression of suicidal ideation or current aggressive/dangerous behaviour
- Relevant current or past hematologic, hepatic, renal, neurologic or other medical disorder that, in the opinion of the principal investigator (PI), might interfere with the study
- Pregnant or breastfeeding

#### Withdrawal criteria

Participants may be withdrawn from the research study for the following reasons:

- At their own request
- At the discretion of the investigator
- Participants who meet the exit criteria for transition to psychosis or develop mania
- Experiencing a serious adverse event or moderate to severe adverse drug reaction
- Participants meeting criteria for insufficient adherence, defined as either taking < 75% of trial medication between assessment points at baseline, 1, 3 and 6 months or missing trial medications altogether for ≥ 7 days in any period
- Becoming pregnant
- An antipsychotic being prescribed during the course of the study

### Recruitment procedures

Consenting patients with capacity will be recruited from the community, general practitioner clinics and psychiatric units in Pakistan.

### Instruments

The following instruments will be used:

- *Prodromal Questionnaire-16 (PQ-16)* [23]: A brief self-report screening questionnaire that assesses the presence of attenuated psychotic symptoms, the PQ-16 is a reliable measure and correlates well with the CAARMS. It is a good screening instrument, and the low number of items makes it feasible to screen populations [24].
- *SOFAS* [18]: A global rating of current functioning, this instrument focuses on social and occupational functioning that is independent of the overall severity of the individual’s psychological symptoms [25].
- *CAARMS* [17]: A semi-structured interview for the identification of individuals at increased risk of developing a first-episode psychotic disorder, it shows good to excellent concurrent, discriminant and predictive validity and excellent inter-rater reliability [17].
- *CogState* [19]: This instrument comprises a number of individual tasks, each designed to test a specific area of cognition. The test battery is culturally neutral and user-friendly, and it covers all necessary cognitive domains. It comprises a range of computerised cognitive tasks able to measure baseline and change in all cognitive domains, and it shows good reliability and validity [26].
- *MADRS* [20]: MADRS is an index of depression administered by a trained clinician who assigns a severity rating for symptoms of depression on the basis of a personal interview. The scale has demonstrated strong reliability, validity and sensitivity to change [27].
- *Schizotypal Personality Questionnaire* [26]: A self-report measure of schizotypal personality disorder, this questionnaire incorporates nine schizotypal features. The scale consists of 74 yes/no items and has been shown to have strong reliability and validity [28].

We will use a structured rating scale to capture information on side effects. All the instruments have been translated into the Urdu language.

### Study procedure

**Fig. 1:**
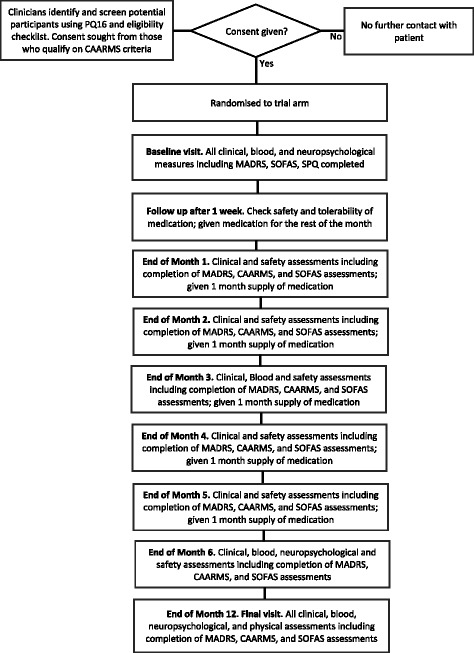
Study flowchart outlining the timetable of study procedures. *CAARMS* Comprehensive Assessment of At Risk Mental States, *MADRS* Montgomery-Åsberg Depression Rating Scale, *PQ-16* Prodromal Questionnaire-16, *SOFAS* Social and Occupational Functioning Assessment Scale, *SPQ* Schizotypal Personality Questionnaire

**Fig. 2:**
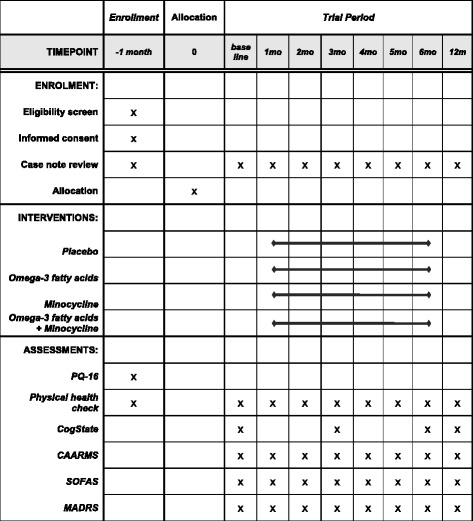
Schedule of enrolment, interventions and assessments. *CAARMS* Comprehensive Assessment of At Risk Mental States, *MADRS* Montgomery-Åsberg Depression Rating Scale, *PQ-16* Prodromal Questionnaire-16, *SOFAS* Social and Occupational Functioning Assessment Scale

The schedule of visits and study procedures is outlined in Fig. 1 and in the Standard Protocol Items: Recommendations for Interventional Trials (SPIRIT) diagram (Fig. 2) and a completed SPIRIT checklist is given in Additional file 1.

#### Consent visit

A trained research assistant (RA) will visit potential participants at their homes or places convenient to them and confirm their suitability for inclusion by screening them using the inclusion/exclusion criteria, confirming consent, and pregnancy testing, if appropriate. The RA will arrange for a doctor to complete checklists for medical history, physical examination and haematological investigations. Participants will also complete the MADRS. Random assignment to the treatment arms will be stratified using the MADRS (total score cut-off ≥ 21) because depressive symptoms may affect illness progression. A computer-generated random sequence based on a block randomised design (with varying block sizes of 2, 4, 6 and 8 in order to reduce predictability [29]) will be administered by an independent third party until all study data are collected and verified. Participants, parents and those involved in administering interventions, assessing outcomes, data entry and/or data analysis will be blind to group assignments. All eligible participants will be randomised to one of the four study arms and allocated a unique identification number, and they will commence study drug treatment within 1 week of providing consent.

#### Baseline visit

The baseline clinical and neuropsychological measures will be completed. The participants will be given a 1-week supply of the study medication appropriate to the trial arm to which they have been randomised; a study information sheet explaining that they are in a clinical study; and the telephone numbers of the senior research clinicians and the clinical trial office, as well as the name of the local PI. In accordance with International Conference for Harmonisation good clinical practice guidelines, copies of all the above will be placed in the individuals’ records along with contact names and telephone numbers.

#### Follow-up visits

The first follow-up visit will occur after 1 week of treatment with the trial medication to check for safety and tolerability of the treatment initiated. At the first follow-up visit, the participant will also receive sufficient trial medication for the rest of the month. Follow-up visits will be conducted at 1-month intervals for the 6-month duration of the intervention and at a final visit 6 months after the end of the intervention (12 months after baseline). During the monthly visits, along with clinical and safety assessments, the participants will complete the MADRS, CAARMS and SOFAS assessments, and they will also receive their study medication. The RA will collect and deliver these to the participant. This will give the research team the opportunity to closely monitor the participant’s physical and mental health, side effects and adherence (by pill count). Female participants with childbearing capacity taking part in the clinical trial will take a pregnancy test regularly. If they should become pregnant during the study, they will be withdrawn from the study.

#### Final visit

At this visit all clinical, neuropsychological and physical assessments will be completed. At all visits the participants’ cumulative clinical drug treatment will be updated from case notes.

### Data management

Data will be collected by trained RAs to maintain data confidentiality and anonymisation. A unique trial identity number will be used for participant data collection and processing. Trial documents (consent form, source documents, assessment tools, case report forms) in hard copies will be kept at the study site in locked file cabinets. Electronic data will be encrypted and password-protected. Backup electronic data will be saved at two places on a hard drive and an institution server. Access to data, both in hard copies and in electronic versions, will be restricted, and only designated persons as approved by the site PI will handle the data. A signed duty log will be used every time the data is accessed. After trial completion the PI will be responsible for data archiving and preservation. Any application for data sharing will also be handled by PI, and access to data will be granted with PI permission.

### Intervention

#### Minocycline

Minocycline will be administered orally for a 6-month period. We have chosen to trial 200 mg because this is the dose used in previous trials in early-phase schizophrenia [30, 31]. A titration schedule will be used to minimise attrition. During the first week of the study, participants will receive a 100-mg capsule of minocycline (or matching placebo) daily. If this is tolerated, the participants will receive 200 mg of minocycline (or matching placebo) for the remaining intervention period. If a participant complains of side effects, the treating psychiatrist (blind to the intervention) will be allowed to omit up to the next two doses of medication and continue from the last prescribed dose. If, despite this, the participant is unable to tolerate an increased dose, the dose will be reduced by 50-mg decrements to a tolerable dose of minocycline or matching placebo.

#### Omega-3 fatty acids

Omega-3 fatty acids will be administered orally, and the daily dose of 1.2 g/day is based on trials in an ultra-high risk population [32]. The active treatment will comprise 600-mg capsules containing concentrated marine fish oil. The proposed daily dose of two capsules will provide 720 mg of eicosapentaenoic acid and 480 mg of docosahexaenoic acid.

#### Placebo

Placebo capsules will be manufactured carefully to resemble the study drugs in appearance. The omega-3 fatty acid (in a soft-shell capsule) and minocycline (in a hard-shell capsule) will appear different because of their individual casing, but all participants will take one hard-shell and two soft-shell capsules, with placebo replacing the active comparator as required.

### Research assistant training and inter-rater reliability

RAs in Pakistan are trained in the assessment measures, and inter-rater reliability will be measured throughout the study by local PIs.

### Statistical analysis

Initially the data will be described using summary statistics, and possible between-group differences will be assessed using analysis of variance (ANOVA) and chi-square tests. Analysis in all cases will be by intention to treat. All protocol violations and major deviations will be recorded as they occur and will be included in reports on trial findings. Because the primary outcome is categorical, logistic regression will be used to assess between-group differences, which will be described using ORs and corresponding 95% CIs. If between-group differences in demographic or other factors are observed, we will estimate adjusted ORs. Secondary and subgroup analyses will also use ANOVA and logistic regression along with other standard hypothesis-testing methods, including Cox regression to analyse potential between-group differences in time to onset of psychosis with non-completers and those who do not develop psychosis included as censored observations. A complete statistical analysis plan will be written prior to database lock and subsequently will be discussed and approved by the trial steering committee.

### Discontinuation

The trial will end either when the study period ends or by the recommendation of the drug safety monitoring board if serious untoward events occur. For individual study participants, the intervention will be discontinued if there are adverse effects that warrant this or if the participant requests to exit the study.

### Adverse event reporting

Adverse events, whether related to study conduct and intervention or not, will be recorded. If there is a serious adverse event, immediate notification of collaborating investigators and sites will be undertaken by the site PI. All essential and appropriate measures will be taken to ensure patient safety. If there are safety issues with the investigational medicines, immediate action will be taken across the whole study to prevent others from avoidable harm.

## Discussion

In Pakistan and other low-/middle-income countries, there is no specific TAU for individuals with ARMS. Minocycline and omega-3 fatty acids are inexpensive and widely available, and evidence suggests that they may be effective in delaying or preventing the onset of full psychosis. The compounds may be feasible and beneficial interventions for patients with ARMS, but there is a strong need to assess evidence of their efficacy. Should minocycline and/or omega-3 fatty acids prove to be effective, this will contribute to the growing body of evidence for treatment options in ARMS.

## Trial status

This clinical trial was registered in October 2015 (ClinicalTrials.gov, NCT02569307), and participants are being recruited until the end of 2017.

## Additional file

Additional file 1: SPIRIT 2013 checklist: recommended items to address in a clinical trial protocol and related documents. (DOCX 49 kb)

## Abbreviations

ANOVA: Analysis of variance
ARMS: At-risk mental state
CAARMS: Comprehensive Assessment of At Risk Mental States
CBCM: Cognitive behavioural case management
CBT: Cognitive behavioural therapy
MADRS: Montgomery-Åsberg Depression Rating Scale
PANSS: Positive and Negative Syndrome Scale
PI: Principal investigator
PQ-16: Prodromal Questionnaire-16
PUFA: Polyunsaturated fatty acid
RA: Research assistant
SOFAS: Social and Occupational Functioning Assessment Scale
SPIRIT: Standard Protocol Items: Recommendations for Interventional Trials
SPQ: Schizotypal Personality Questionnaire
TAU: Treatment as usual
